# Ocular Findings in Siblings With Alagille Syndrome: A Report of Two Cases

**DOI:** 10.7759/cureus.95312

**Published:** 2025-10-24

**Authors:** Ricardo A Murati Calderon, Julian A Menendez Sepulveda, Natalio Izquierdo

**Affiliations:** 1 Ophthalmology, University of Puerto Rico, Medical Sciences Campus, San Juan, PRI; 2 Medicine, University of Puerto Rico, Medical Sciences Campus, San Juan, PRI; 3 Surgery, University of Puerto Rico, Medical Sciences Campus, San Juan, PRI

**Keywords:** alagille syndrome, anterior segment dysgenesis, autosomal dominant inheritance, genotype-phenotype correlation, ophthalmology

## Abstract

Alagille syndrome (ALGS) is a multisystem disorder, most often caused by *JAG1* variants. Ocular features classically include posterior embryotoxon and optic disc anomalies, but glaucoma is uncommonly reported. We report two Latino siblings with ALGS and glaucoma. They share the previously described *JAG1* c.925G>C (p.Gly309Arg) variant, classified as a variant of uncertain significance (VUS). One sibling exhibited a posterior embryotoxon, accompanied by retinal nerve fiber layer (RNFL) thinning and elevated intraocular pressure (IOP), which responded to topical therapy. The other had a microcornea, as well as iris and chorioretinal colobomas, with asymmetric cupping and elevated IOP, which was also responsive to topical treatment. The concordant phenotype and shared genotype in these siblings highlight a potential clinical significance, suggesting that disruption of the Notch pathway may increase the risk of anterior-segment (mesodermal) dysgenesis and predispose individuals with ALGS to glaucoma. These cases broaden the recognized spectrum of ALGS phenotypes and underscore the importance of targeted glaucoma screening and longitudinal follow-up, particularly in underrepresented populations.

## Introduction

Alagille syndrome (ALGS) is a rare multisystemic disorder characterized by a wide range of clinical manifestations, including ophthalmic findings [1]. Major diagnostic criteria of patients with ALGS include cholestatic liver disease due to bile duct paucity, characteristic facial dysmorphism, cardiovascular anomalies (mainly pulmonary artery stenosis), and renal and skeletal abnormalities, such as butterfly vertebrae [2]. Ocular features in patients with the syndrome include posterior embryotoxon, iris abnormalities, optic disc anomalies, diffuse fundus hypopigmentation, and speckling of the retinal pigment epithelium [1,3]. 

The primary genetic cause of ALGS is mutations in the *JAG1* gene, inherited as an autosomal dominant trait [4]. The *JAG1* gene encodes the jagged 1 protein, a key component of the Notch signaling pathway. Human jagged 1 is the ligand for the receptor notch 1, which is involved in signaling processes [5]. JAG1-Notch signaling regulates the differentiation of endothelial cells into mesenchymal cells, a process known as endothelial-mesenchymal transition [6]. This pathway is vital for forming bile ducts, heart valves, and other key structures, such as the neural-crest-derived anterior segment of the eye, commonly affected in ALGS [7,8]. 

Perturbation of the Notch pathway in the anterior segment has been implicated in the prominence of Schwalbe’s line, known as posterior embryotoxon, abnormal development of the trabecular meshwork and Schlemm’s canal, and various iris anomalies [1]. These alterations provide a biologically plausible mechanism for impaired aqueous outflow and subsequent elevation of intraocular pressure (IOP). In animal models, mutations in *JAG1* have been shown to similarly cause abnormalities and dysgenesis of the trabecular meshwork and iridocorneal angle, supporting this mechanistic link [9]. 

Alagille syndrome affects approximately 1 in 30,000 to 70,000 live births globally and exhibits variable expressivity, complicating diagnosis and management [1]. We report the case of two siblings, 32 and 38 years old, both male, with ALGS who developed glaucoma. The rarity of glaucoma in ALGS suggests it may be under-recognized. Additionally, one sibling has bilateral coloboma. To our knowledge, this occurrence of glaucoma has been infrequently reported in patients with the syndrome [3,10]. This case report aims to add to the limited literature on this association, offering insight into potential mechanisms linking ALGS and glaucoma.

## Case presentation

Case 1

A 32-year-old Latino male patient with a medical history of mild cognitive impairment, hearing loss, and epilepsy was evaluated at our genetic clinic after referral from a retina specialist due to findings of rod-cone dystrophy during fundus examination. The patient has a sibling with developmental delay and iris coloboma. Upon examination, he exhibited craniofacial features, such as micrognathia and gingival disorders, consistent with dysmorphic features. He reported a past ocular history of nyctalopia since early childhood. The patient's extraocular history included bile duct paucity with chronic cholestasis since early childhood. A comprehensive metabolic panel repeatedly demonstrated elevated total/direct bilirubin, as well as alkaline phosphatase, with preserved renal function; complete blood counts were within normal reference ranges. Cardiovascular history was notable for valvular heart disease, with clinic documentation of palpitations and an auscultated murmur; prior diagnoses include aortic valve stenosis and mitral valve prolapse. 

**Figure 1:**
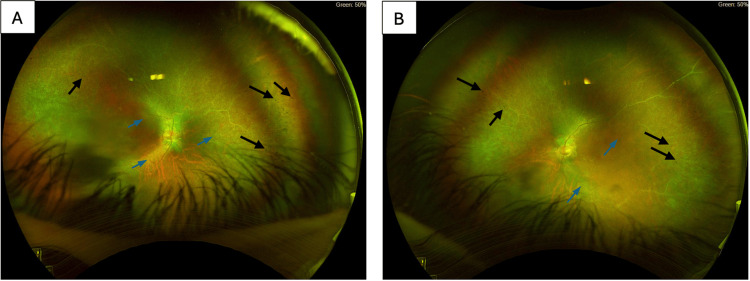
Ultra-widefield fundus photographs of Case 1 (A) Right eye (OD) and (B) left eye (OS) showing optic disc cupping with temporal pallor, diffuse arteriolar attenuation (blue arrowheads), and mid-peripheral bony spicule pigmentation (black arrowheads).

Upon initial comprehensive ophthalmic evaluation by at least one of the authors (NI), the patient had a best-corrected visual acuity of 20/80 in the right eye (OD) and 20/200 in the left eye (OS). IOPs measured by applanation tonometry were 27 mmHg in both eyes (OU). The central corneal thickness was 552 μm OD and 550 μm OS. An external evaluation demonstrated alternating nystagmus in OU. Upon slit-lamp examination, the patient had a prominent Schwalbe's line and a smooth velvety iris without iridodonesis in OU. A dilated fundus examination revealed bilateral optic disc cupping, temporal pallor, arteriolar narrowing, and bony spicules in the mid-periphery (Figure 1A-1B). 

**Figure 2:**
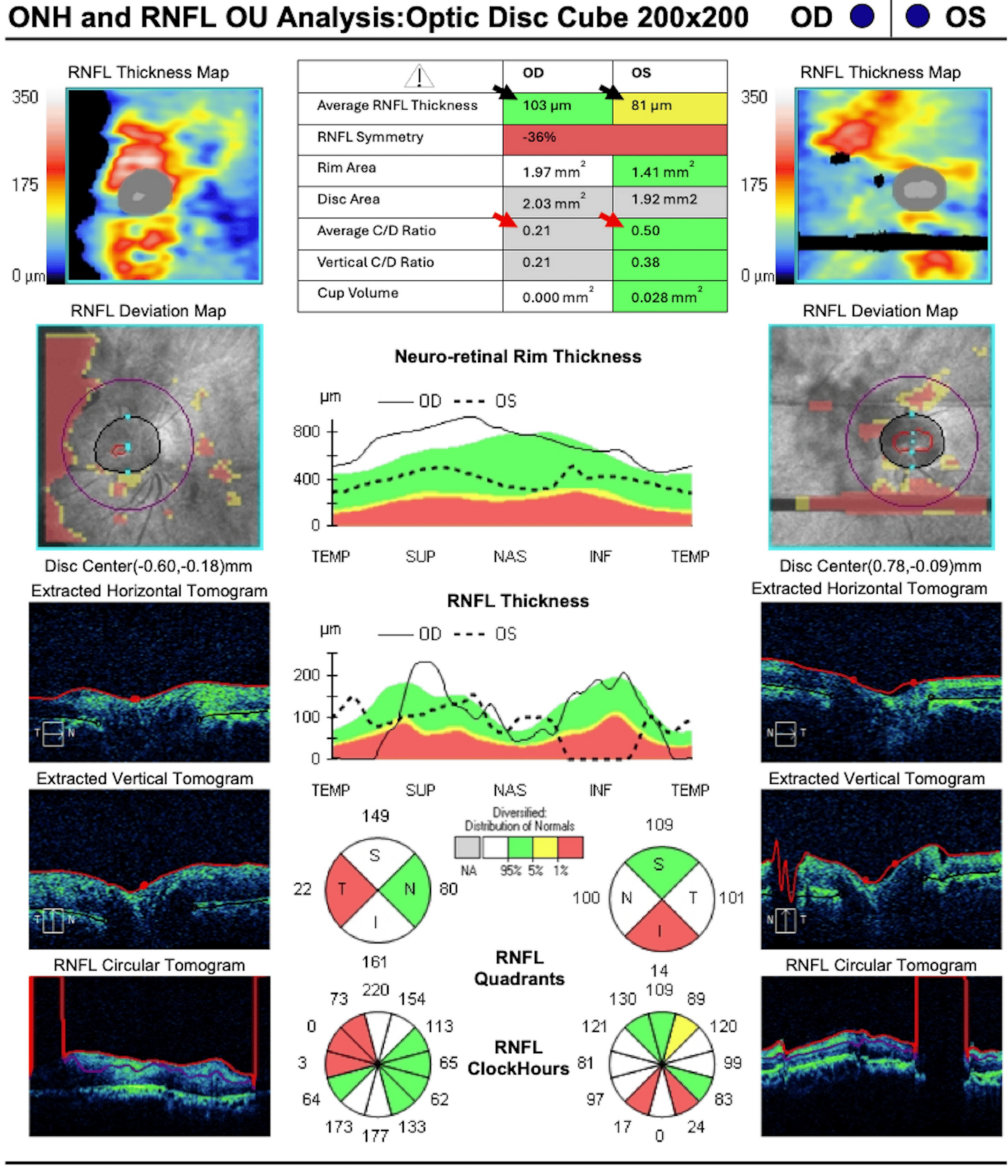
Spectral-domain OCT of the optic nerve in Case 1 Optic nerve OCT demonstrated asymmetry between both eyes in both RNFL (black arrowheads) and the average cup-to-disc ratio (red arrowheads). ONH: optic nerve head, OCT: optical coherence tomography, RNFL: retinal nerve fiber layer, C/D ratio: cup-to-disc ratio, Temp: temporal, Sup: superior, Nas: nasal, Inf: inferior, OD: right eye, OS: left eye, OU: both eyes.

Spectral-domain optical coherence tomography (OCT) of the optic nerve (Cirrus; Carl Zeiss Meditec AG, Dublin, OH, USA) (Figure 2) showed both asymmetric cup-to-disc ratio and retinal nerve fiber layer thickness (RNFL). The average cup-to-disc ratio was 0.21 OD and 0.50 OS. The RNFL thickness was 103 µm OD and 81 µm OS. Neuro-retinal rim thickness varied across all quadrants when comparing OU. It is of note that there are significant segmentation errors in OU, which delineate the limitations of the technology to provide accurate scans and readings in the setting of an uncooperative or poorly fixating patient. Due to the patient's cognitive impairment, an adequate Humphrey visual field test could not be completed. 

A diagnosis of glaucoma was reached. The patient was initially started on brimonidine 0.1% twice daily, but he developed periocular pruritus and hyperemia. Therapy was therefore switched to dorzolamide 2% ophthalmic solution administered every eight hours in OU. At the time of the last follow-up, one year after the initial evaluation, the patient’s IOP had reduced to 16 mmHg in OU, and the medication was reported to be well tolerated. His visual acuity remained stable at 20/80 OD and 20/200 OS, with no noticeable progression in the cup-to-disc ratio, as noted by stable cupping of 0.3 OD and 0.5 OS, respectively.

Genetic testing was conducted using a saliva sample, and a next-generation sequencing (NGS) diagnostic test (Invitae Inherited Retinal Disorders Panel; Invitae Corporation, San Francisco, CA, USA) was performed to evaluate over 330 genes associated with inherited retinal genetic diseases. NGS and deletion and duplication analysis done using the Invitae Panel showed a heterozygous mutation in the *JAG1 *gene. The variant was c.925G>C (p.Gly309Arg). This mutation was classified as a variant of uncertain significance (VUS). 

Case 2

A 38-year-old Latino male patient with developmental delay, bilateral cryptorchidism, and bilateral iris coloboma presented to the clinic for routine ophthalmologic evaluation. He sought this evaluation after his younger sibling's examination showed genetic results suggestive of ALGS. He displayed dysmorphic facial features documented since infancy. The patient's extraocular history included motor neuron disease and a seizure disorder. Evaluation by an adult congenital heart disease specialist identified a possible patent foramen ovale and cor triatriatum, with transthoracic echocardiography documenting aortic and tricuspid valve regurgitation. Unfortunately, he has not yet been evaluated by a gastroenterologist to further characterize potential hepatic involvement. Available laboratory data show a comprehensive metabolic panel to be essentially unremarkable and a complete blood count within normal reference ranges. 

Upon comprehensive ophthalmologic evaluation, his best-corrected visual acuity was 20/400 OD and 20/70 OS. IOP measured by Goldmann tonometry was 23 mmHg in OU. Slit-lamp examination revealed microcorneas with horizontal diameters of 10.0 mm in OU. The anterior chamber was deep and quiet, with no signs of inflammation. The patient had iris coloboma and ectopia lentis with cortical cataracts in OU. Dilated fundus examination revealed asymmetric optic disc cupping of 0.21 OD and 0.35 OS, with an inferior chorioretinal coloboma. The RNFL was thinned in OU; blood vessels emanated from the superior border of the coloboma. Additionally, peripheral pigmentary changes were noted in OU.

**Figure 3:**
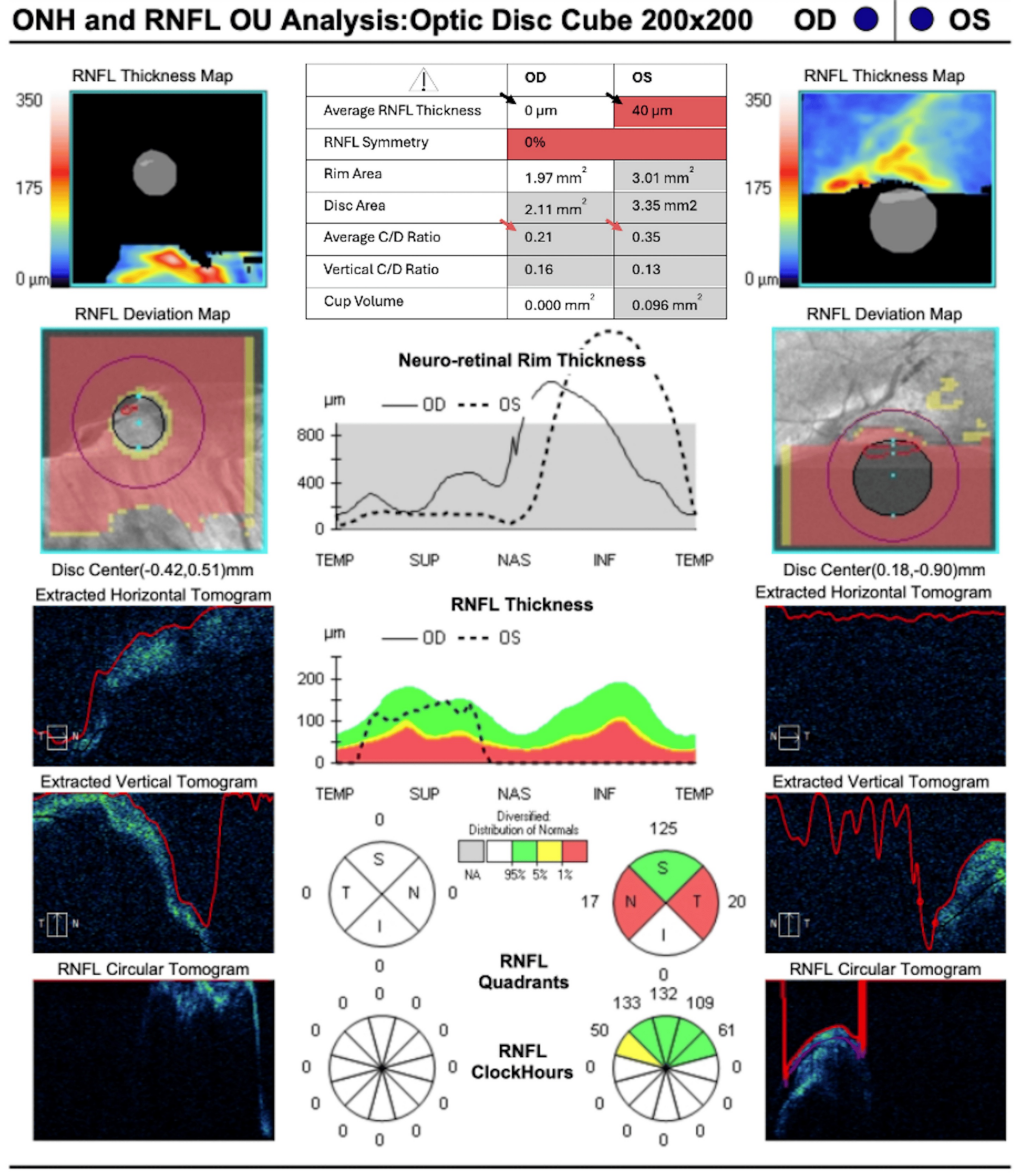
Spectral-domain OCT of the optic nerve in Case 2 Optic nerve OCT scan quality was very low (signal strength 1/10) with decentration and vignetting. Automated peripapillary RNFL segmentation failed over large regions, with the available data suggesting relative rim thinning (black arrowheads) and larger cupping in the left eye (red arrowheads). ONH: optic nerve head, OCT: optical coherence tomography, RNFL: retinal nerve fiber layer, C/D ratio: cup-to-disc ratio, Temp: temporal, Sup: superior, Nas: nasal, Inf: inferior, OD: right eye, OS: left eye, OU: both eyes.

Spectral-domain OCT of the optic nerve (Figure 3) was attempted but yielded very low signal strength, with indeterminate peripapillary RNFL segmentation across large regions, resulting in non-physiologic global values and incomplete rim plots. These limitations likely reflect the patient's limited cooperation, related to developmental delay and cognitive impairment. 

Subsequently, a diagnosis of** **glaucoma was reached, and the patient was started on brimonidine 0.1% ophthalmic eye drops two times daily in OU. At the one-year follow-up visit, the IOP was reduced to 13 mmHg OD and 12 mmHg OS. The patient's condition has remained stable to date. 

An NGS diagnostic test evaluating over 330 genes for variants associated with genetic disorders showed a heterozygous mutation in the *JAG1* gene, with VUS c.925G>C (p.Gly309Arg). 

Further NGS analysis of both parents showed no mutations in the mother. However, the father was found to have the same heterozygous mutation in the *JAG1* gene, with VUS c.925G>C (p.Gly309Arg). 

## Discussion

ALGS, first described by French hepatologist Daniel Alagille in 1969, is characterized by cholestasis, distinctive facial features, and ocular anomalies [11]. Our patient's micrognathia is compatible with the typical facial features of patients with ALGS, further supporting the diagnosis. 

Previous studies by Hingorani et al. have shown that most patients with the syndrome have good visual acuity despite structural ocular anomalies [3]. In our case, both patients had significant visual impairment in both eyes, contrasting with the prior reports described. Severe visual impairment, as found in our patients, may be due to glaucoma, leading to optic neuropathy and visual field loss. 

Da Palma et al. described that up to 90% of patients with ALGS have ocular manifestations, including posterior embryotoxon and iris and optic disc anomalies [1]. Potamitis and Fielder reported an episode of acute angle closure glaucoma in a patient with ALGS [10]. Patient 1 had a prominent Schwalbe's line, smooth velvety iris, and increased IOP compatible with mesodermal dysgenesis leading to secondary glaucoma. Patient 2 had bilateral iris coloboma. These findings are consistent with glaucoma in the setting of anterior-segment dysgenesis in ALGS, a mechanism that has been under-reported.** **

In patients with ALGS, elevated IOP may arise from several mechanisms beyond primary open-angle disease. First, steroid-induced ocular hypertension should be excluded, as topical, periocular, or systemic corticosteroids, used for other manifestations or concurrent conditions, can precipitate IOP elevation. However, careful medication reconciliation in our cases helped rule out this etiology. Second, angle-dysgenesis-related glaucoma is biologically plausible in ALGS, given the involvement of the Notch pathway in anterior-segment development. The presence of associated findings, such as posterior embryotoxon, microcornea, and iris anomalies, can support this mechanism. Additional considerations include the risk of angle closure from crowded anterior segments and inflammatory secondary glaucoma when uveitis or ocular inflammation is present. In our case, targeted evaluation with gonioscopy, anterior-segment OCT, and review of steroid exposure helped distinguish these distinct entities.

Ocular abnormalities, such as posterior embryotoxon or a prominent Schwalbe's line, as well as iris abnormalities like coloboma, can all contribute to impaired drainage of aqueous humor. These defects may contribute to increased IOP, leading to secondary glaucoma in the context of mesodermal dysgenesis. Thus, understanding the developmental pathway of these ocular structures provides insight into how *JAG1* mutations may predispose ALGS patients to glaucoma. 

While glaucoma is not a primary ocular manifestation associated with ALGS, this report provides important information that highlights a potential link between the syndrome and this ocular condition. In Da Palma’s patient cohort (23 patients, 44 eyes), IOPs ranged from 8 to 22 mmHg, with a mean and median of 16, and only one eye was higher than 21 mmHg [1]. This contrasts with our two-patient series, with pressures (pooled across OU and visits) ranging from 12 to 27 mmHg, with a mean of 19.6 mmHg, and a median of 19.5 mmHg. This difference could be explained by phenotypic variance between specific alleles, but the patient's age at the time of evaluation could be a factor, too. Da Palma’s cohort’s mean and median ages were 22 and 16 years, respectively, while ours were 35 for both mean and median [1]. This case underscores the importance of early screening for glaucoma in patients with ALGS, especially those with anterior-segment dysgenesis. Regular ophthalmologic evaluations and multispecialty care are crucial for the well-being of these patients. 

Previous studies have shown numerous variants in the *JAG1* gene associated with the syndrome. In this family, both affected siblings carry the previously described *JAG1* variant c.925G>C, currently classified in ClinVar as a VUS. In the gnomAD database, this variant is scarce with a global allele frequency of 3.78 x 10^-5^ and no reported homozygotes, aligning with expectations for rare monogenic retinal disorders. Although its pathogenicity is unresolved, its co-occurrence with a classic Alagille phenotype in both siblings supports potential clinical relevance. Given *JAG1*'s role in Notch signaling and mesodermal development of anterior segment structures, this variant could plausibly contribute to mesodermal dysgenesis and to glaucoma. Its identification in a Latino family also adds representation from an underreported population.

Studies by Li et al. discuss the role of the Notch signaling pathway, involving the *JAG1* gene, in the differentiation of endothelial cells into mesenchymal tissue, which is essential for the development of various ocular structures, including the cornea [12]. Although their focus was primarily on corneal development, the broader involvement of Notch signaling in the differentiation of mesoderm-derived ocular structures may help explain how *JAG1* mutations could potentially lead to defects in the trabecular meshwork, which is crucial for the regulation of aqueous humor outflow. 

This case addresses an important gap in the literature by highlighting the potential role of *JAG1* mutations in anterior segment dysgenesis and their implications in glaucoma development. Further research is essential to establish more definitive associations. Larger cohort studies and population-based analyses are needed to delineate this connection better. Limitations of the study include its reliance on two siblings.

To our knowledge, there is a lack of large-scale studies linking *JAG1* mutations and glaucoma in ALGS, with most research focusing on anterior segment anomalies. It is imperative that future studies investigate the mechanisms underlying glaucoma in ALGS and that multidisciplinary management be established for these patients.

## Conclusions

Our case expands the phenotypic spectrum in patients with ALGS. Identification of a VUS in the *JAG1* gene in both cases emphasizes the need for continual genotype-phenotype correlation in this syndrome. The observation of a prominent Schwalbe's line in Patient 1, iris coloboma in Patient 2, and glaucoma in both patients suggests that clinicians should monitor for glaucoma in ALGS patients. Early detection and intervention are crucial for vision preservation, underscoring the clinical importance of proactive glaucoma screening in this population.
